# Application of UAV Remote Sensing in Monitoring Water Use Efficiency and Biomass of Cotton Plants Adjacent to Shelterbelt

**DOI:** 10.3389/fpls.2022.894172

**Published:** 2022-06-16

**Authors:** Bin Ma, Qijie Wang, Bing Xue, Zhenan Hou, Yan Jiang, Wenyue Cai

**Affiliations:** ^1^Agricultural College, Shihezi University, Shihezi, China; ^2^National Climate Center, China Meteorological Administration, Beijing, China

**Keywords:** AquaCrop model, UAV remote sensing, water use efficiency, biomass, shelterbelt

## Abstract

Tree shelterbelts are crucial for maintaining the ecological environment of oasis, but they may also compete for soil water with adjacent crops, affecting crop yields. To evaluate the impacts of the shelterbelt on water use efficiency (WUE) and normalized water productivity (WP) of adjacent cotton plants, the biomass (B) and WUE of cotton with different distances from the shelterbelt (0.1H, 0.5H, 1H, 2H, and 3H; average tree height = 15 m [H]) were estimated based on unmanned aerial vehicle (UAV) remote sensing data combined with the FAO crop water response model AquaCrop. Besides, the accuracy and universality of the estimation method were also evaluated. The results showed that the method based on UAV remote sensing data and AquaCrop can accurately estimate the impact range and intensity of shelterbelt on WUE, water consumption, and B of adjacent cotton plants. Fierce water competition between shelterbelt and cotton was detected within 0.1H−1H, and the competitiveness of the shelterbelt was weaker in the plots >1H than in the 0.1H−1H. The B, actual evapotranspiration (T_c_), and WUE of cotton at 0.1H decreased by 59.3, 48.8, and 23.6%, respectively, compared with those at 3H, but the cotton plants at 2H and 3H were completely unaffected by the shelterbelt. Besides, the B estimated based on WP (root mean square error [RMSE] = 108 g/m^2^, d = 0.89) was more accurate than that estimated based on WUE (RMSE = 118 g/m^2^, d = 0.85). This study clarifies the inter-species competition for soil water between crops and shelterbelts under drip irrigation in oases in China.

## Highlights

1. Combining the AquaCrop model with spectral and measured data to estimate cotton growth and the continuous water consumption.
2. Quantitatively evaluate the impacts of shelterbelts on the productivity and WUE of cotton field.
3. Selected a higher accuracy and applicability water use efficiency model for Xinjiang oasis area.
4. Estimated the spatial distribution of WUE and biomass of the cotton field adjacent to shelterbelts.

## Introduction

Tree shelterbelts have been widely used as ecological barriers for an oasis. It could improve the ecological environment and agricultural productivity and could ensure the living space of human beings (Sagrario et al., 2010; Xue et al., 2019; Zhao et al., 2020). Water resources are very limited in an oasis, while the deep-rooted and leafy shelterbelts consume a lot of water. Studies have found that shelterbelts may lead to or exacerbate water resource conflicts (Xi et al., 2013; Fu et al., 2016, 2020), and decrease water use efficiency (WUE) and yield of adjacent crops (Li et al., 2010; He et al., 2016; Liu et al., 2020). Water is the most important factor influencing the growth of plants in an oasis (Ong et al., 2002; Wang et al., 2018; Zhang et al., 2022). How to accurately estimate the impacts of the shelterbelts on the productivity and WUE of adjacent crops is a key issue to effectively utilize limited water resources and improve crop yields in an oasis. However, at present, there are few studies on the impacts of the shelterbelt on the WUE and productivity of adjacent crops in arid areas, such as Xinjiang, China (Sang et al., 2015; Lian et al., 2021).

In previous studies, traditional model simulation and field measurement are widely used to estimate the WUE and biomass (B) of plants. However, in agroforestry systems, the complexity of measurement and data processing of various parameters is greatly increased. In recent years, with the rapid development of remote sensing, the model estimation based on remote sensing data provides an efficient means for the estimation of water consumption, WUE, and B of crops in agroforestry ecosystems (Sadras et al., 2014; Thorp et al., 2018). However, the resolution of satellite remote sensing images is low, which lowers the estimation accuracy (Jin et al., 2012). Unmanned aerial vehicle (UAV)-based remote sensing has the advantages of temporal continuity and high resolution and is less influenced by weather conditions, compared to satellite remote sensing. Therefore, it has a great potential in improving the estimation accuracy of WUE and B of crops (Huang et al., 2018; Ji et al., 2019; Han et al., 2021). Niu et al. (2019) and Bendig et al. (2015) obtained high estimation accuracy of crop B by using UAV remote sensing images to construct normalized difference vegetation index (NDVI). Wang et al. (2013) also constructed the models using UAV images and accurately estimated the primary productivity and WUE of crops. However, the water consumption of crops is constantly changing (Thorp et al., 2018). Previous studies have estimated the B or WUE of crops at a certain time or period based on remote sensing data, which could not clarify the water use of crops during the entire growth period (Dimitrios et al., 2018). It is worth noting that Steduto et al. (2009) constructed a crop water response model based on soil water data and crop canopy remote sensing data, which accurately determined the response of crop B to water by calculating WUE. Zhang et al. (2019) combined the FAO crop water response model (AquaCrop) with UAV-based spectral data of crops and achieved precise monitoring of crop growth, B accumulation, and continuous water consumption (White et al., 2012). However, there are currently few studies using this method to monitor agroforestry systems in arid regions.

In recent years, with the widespread application of drip irrigation in the arid areas in Xinjiang, China, it is difficult for tree shelterbelts to get water supply, leading to tree roots extending to adjacent farmland to obtain water. This severely affects the WUE and productivity of adjacent crops. Zhu et al. (2015) showed that under drip irrigation conditions, the horizontal influence range of tree roots in farmland was up to 10–12 m, leading to a 30% loss of cotton yield. At present, some small-area simulation experiments have been conducted to determine the effect of drip irrigation on cotton productivity in agroforestry systems. However, the estimation accuracy tends to be lower when their methods are applied to large areas.

Therefore, in this study, a new method that combines UAV-based remote sensing data with the AquaCrop model was proposed to estimate the B and WUE of cotton plants at different distances from adjacent shelterbelt in an oasis. This study aimed to (i) quantitatively evaluate the impacts of shelterbelt on B and WUE of adjacent cotton plants under drip irrigation, (ii) clarify the accuracy of the AquaCrop model in estimating crop B, actual transpiration (T_c_), and WUE, and (iii) select a highly accurate and universal model for monitoring the growth, yield, and continuous water consumption of crops adjacent to shelterbelt in an oasis. This study clarifies the water competition between shelterbelt and adjacent crops under drip irrigation, and provides a reference for formulating irrigation schemes to improve the WUE of crops in an oasis.

## Materials and Methods

### Approach to the Study

**Figure 1:**
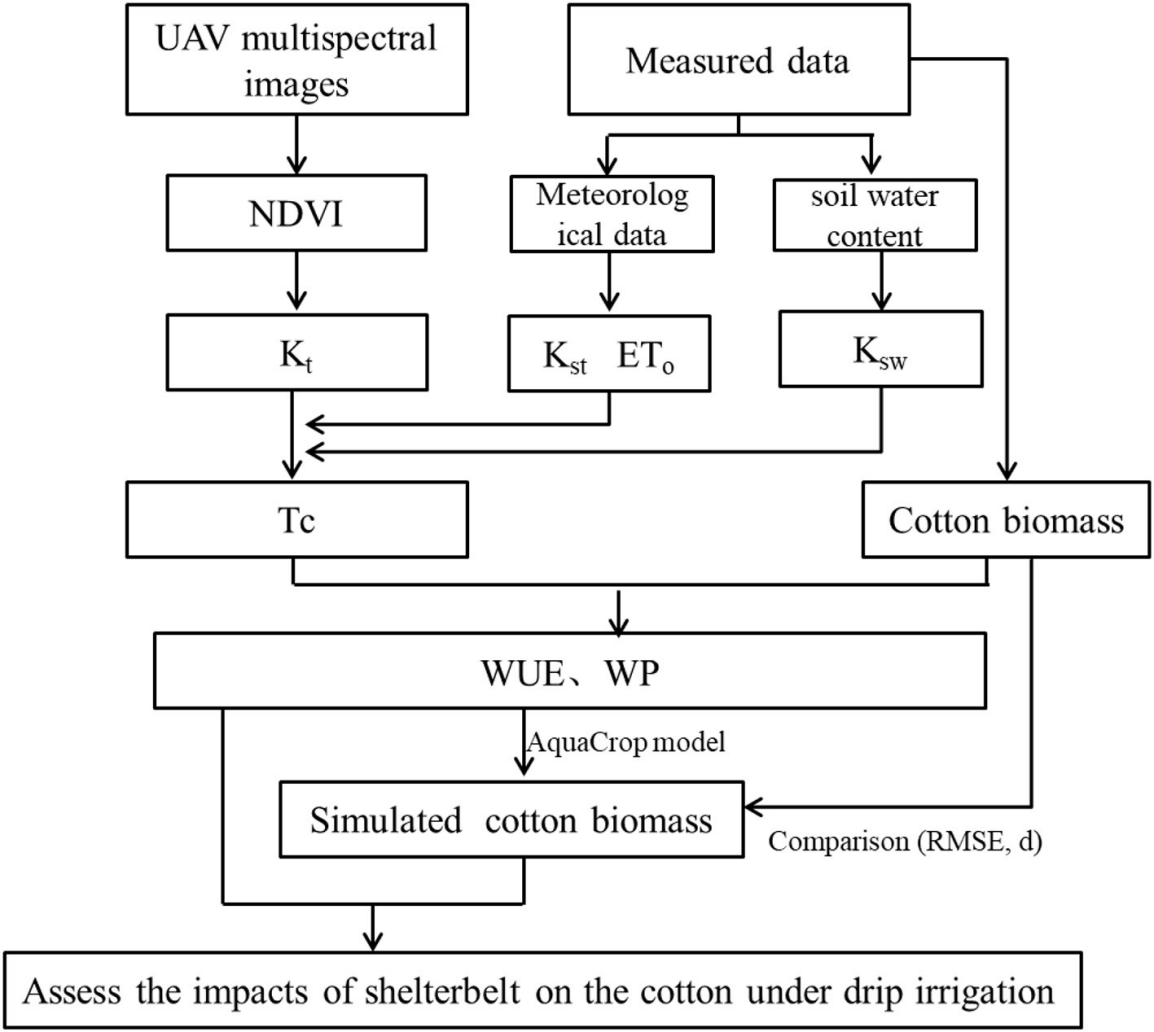
Flow chart of estimation of water use efficiency by constructing a model based on the unmanned aerial vehicle based remote sensing.

Field experiments were carried out in 2021. The growth of the cotton was monitored from planting till harvest, and the weather and soil water content data during the whole growing season were collected to construct the AquaCrop model. First, the transpiration coefficient (K_t_) was estimated using the NDVI (Han et al., 2021) based on a calibrated linear equation (Campos et al., 2017). Then, combined with the meteorological data and soil water content, T_c_ in the whole growth period for each sampling plot was estimated. Through the above steps, WUE and normalized water productivity (WP) (g/m^2^) were fitted using measured B and T_c_ of the sampling plots at different distances. Finally, the simulated B was obtained using the fitted WUE and WP and measured B. Model accuracy was verified by comparing simulated B with measured B. Statistical analysis of measured results and simulated results was conducted to assess the impacts of shelterbelt on cotton B and WUE under drip irrigation in an oasis. Figure 1 shows the overall process of this study.

### Field Experiments

#### Experimental Site and Design

The experiment was conducted in 150 Tuan (44°59'7”N, 86°8'56”E), Shihezi, Xinjiang, China, on the southern edge of the Junggar basin in 2021. The soil is sandy loam. The water holding capacity of the 0–100 cm soil layer was 25.4%, the wilting coefficient was 11.2%, and the soil bulk density was 1.35 g/cm3 (Wu et al., 2021).

The experimental site is rectangular (293 m × 50 m) (Figure 1). The cotton variety “Xinluzao 46” was planted, with a row spacing of 0.66 m and a plant spacing of 0.10 m. Drip irrigation was employed, and irrigation was performed 11 times during the entire growth period of cotton. The irrigation amount per time was 675 m^3^/ha. The date of emergence was April 26 and the date of defoliation was September 16. The tree species of the shelterbelt was *Populus alba* var. The average tree height (H) was 15 m, the tree density was 3 × 3 m, and the root depth was 2–4 m. The distance between shelterbelt and cotton field was 0.5 m.

**Figure 2:**
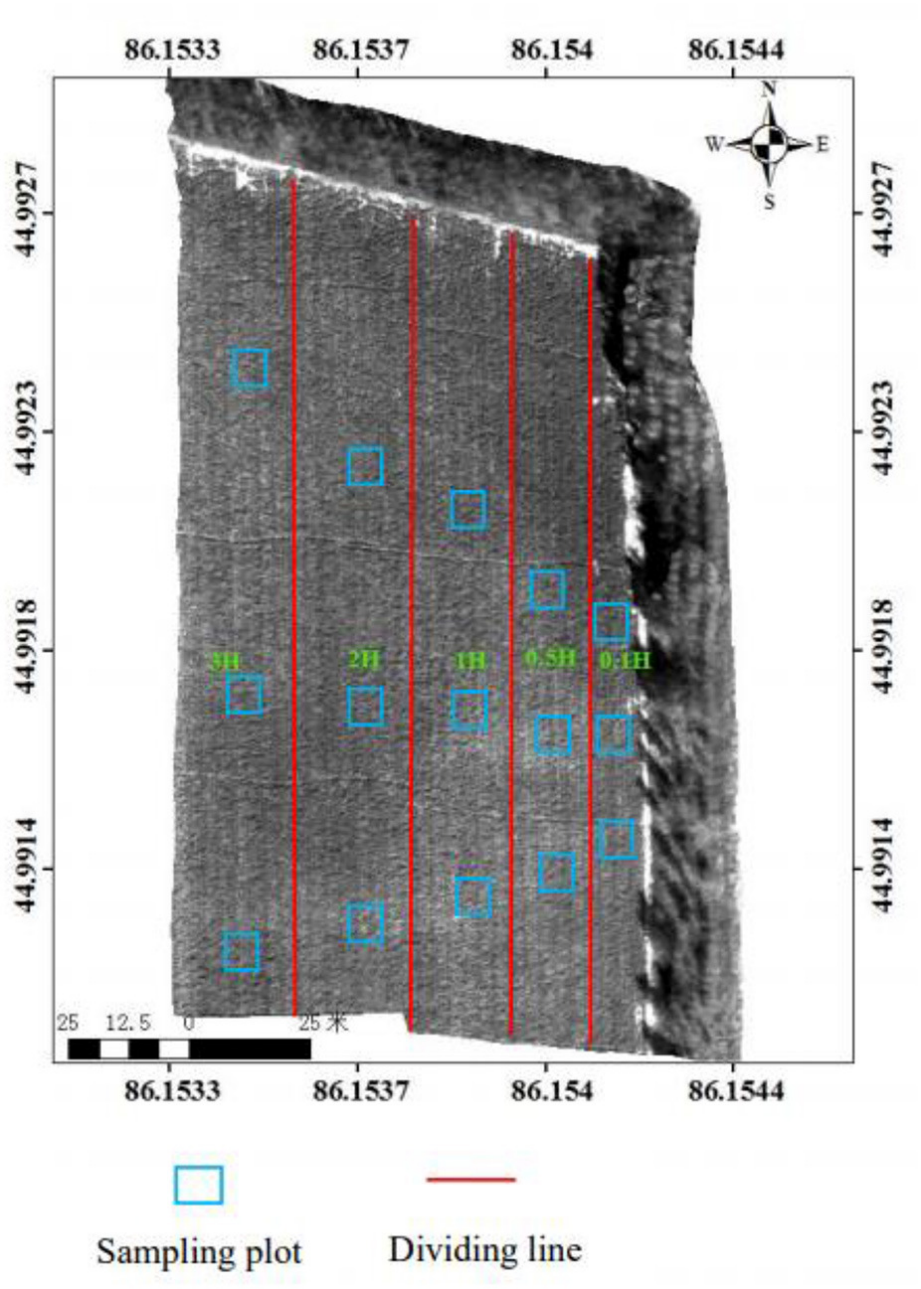
Experimental site.

Three sampling plots (1 × 1 m) were selected at each distance of 0.1H, 0.5H, 1H, 2H, and 3H from the shelterbelt (a total of 15 sampling plots) (Figure 2). The precipitation amount and field managements were the same for the plots in the whole growth period.

#### Collection of Meteorological Data

Meteorological data collected by the Mosuowan Weather Station (2 km away from the experiment site) were used in this study, and the data were obtained from the Information Center of the National Meteorological Administration.

#### Soil Water Content

The 0–20, 20–40, 40–60, 60–80, and 80–100 cm soil layers of the sampling plots were sampled separately, and the average soil water content of the five soil layers was considered as the soil water content of the sampling plot. From the seedling stage, soil sampling was performed every 15–20 days. The soil water content was determined using the drying method. Additional measurements were made before and after irrigation and after rainfall (Shao and Wu, 2019; Qiao et al., 2021).

#### Determination of Cotton Biomass

After each data acquisition, three cotton plants were randomly selected in each sampling plot on May 26, June 10, June 24, July 12, August 2, August 16, August 28, and September 15, for aboveground B determination. Plant samples were dried at 105°C for 30 min, and then dried at 80°C to constant weight. Finally, the dried samples were weighed.

### Data Acquisition

The UAV equipped with a fixed-wing EBEE frame, a Sequoia multispectral camera (Switzerland), and a multispectral image acquisition system was used for spectral data acquisition. The camera has a light intensity sensor and a calibration plate and can acquire the spectra at 560 nm (green light, G), 668 nm (red light, R), 717 nm (red edge, RE), and 840 nm (near-infrared, NIR).

The image acquisition was performed at 11:00–13:00 since the seedling stage of cotton, with an interval of 15 days. The flight altitude of the UAV was 100 m. The resolution was 11.79 cm/pixel. The flight routes were consistent, and the course overlap and side overlap were 75 and 80%, respectively. The UAV images collected each time were spliced by Pix4D Mapper (Pix4D, Switzerland). The splicing process mainly included the imports of images and coordinates of ground control points, generation of point clouds, radiometric correction, digital surface models, orthophoto images, and NDVI distribution maps (Bannari et al., 1996). The NDVI distribution maps were clipped using ENVI 5.3 (Exelis Visual Information Solutions, USA) based on the shp files of the 15 sampling plots to extract the NDVI values.

### Estimation of Cotton Biomass Based on UAV-Based Multispectral Remote Sensing and AquaCrop Model

#### Biomass Estimation Based on WUE

The modeling based on WUE (kg/m^3^) is the initial approach to estimate crop growth status. The Aquacrop model estimates the crop B by calculating the product of the total T_C_ from sowing to harvest and WUE. WUE is the dry matter accumulated by the crop consuming a unit of water (Briggs and Shantz, 1913), as given in the following equation:


(1) $$\begin{array}{r} B=WUE\times \overset{t_{1}}{\underset{t_{0}}{\sum }}T_{c} \end{array}$$

where B is the increase in cotton B from t_0_ to t_1_ (g/m^2^), t_0_ is the emergence time of cotton, t_1_ is the measuring time of B, T_c_ is the actual evapotranspiration (mm) from sowing to harvest [Equation (3) and Equation (4)], and WUE is the slope of the relationship between measured B and T_c_.

#### Biomass Estimation Based on WP

Because the essentially constant (or linear) relationship between B and T_c_ (WUE) is easily affected by climate and season, it is necessary to standardize T_c_ for different climates. Normalization of WUE using ET_0_ is a way to normalize optimal WUE (WP) (Steduto et al., 2007, 2012; Han et al., 2021). The relationship between B and the cumulative value of the T_c_/ET_0_ ratio is presented in Equation (2) according to the methodology in the AquaCrop model and the FAO-66 manual (Steduto et al., 2012).


(2) $$\begin{array}{r} B=WP\times \overset{t_{1}}{\underset{t_{0}}{\sum }}\frac{T_{c}}{ET_{0}} \end{array}$$

(3) $$\begin{array}{rcl} T_{c} & = & K_{sum}\times ET_{0} \end{array}$$

(4) $$\begin{array}{rcl} K_{sum} & = & K_{t}{\times K}_{sw}{\times K}_{st} \end{array}$$

where WP is the normalized WP (g/m^2^), K_t_ is the transpiration coefficient [Equation (5) and uation (6)], K_st_ is the temperature stress coefficient [Equation (7) and Equation (8)], and K_sw_ is the water stress coefficient [Equation (9)].

#### Parameterization of AquaCrop Model

**(1) Remote sensing of transpiration coefficient (K**_**t**_ **)**

K_t_ is the key to determine T_c_. UAV-based remote sensing can obtain high-resolution cotton canopy information, and continuous K_t_ distribution in farmlands could be obtained by combining it with the empirical model based on NDVI (Campos et al., 2018b). In this study, K_t_ was calculated according to the method of Gonzalez-Dugo and Mateos (2008).


(5) $$\begin{array}{r} K_{t}=K_{t,max}\left[1-{\left(\frac{NDVI_{max}-NDVI}{NDVI_{max}-NDVI_{min}}\right)}^{\alpha }\right] \end{array}$$

where K_t, max_ is the maximum K_t_ when cotton canopy coverage is the largest, NDVI_max_ is the maximum NDVI when cotton canopy coverage is the largest (NDVI_max_ = 0.93), NDVI_min_ is the NDVI based on bare soil (Bannari et al., 1996) (NDVI_min_ = 0.20), and α is the simple linear model, taken as 1 (Han et al., 2021).

The FAO-56 dual crop coefficient method has been widely used to estimate K_t, max_ (Feng et al., 2016). In this study, according to the calculation formula of FAO-56 (Allen et al., 1998), K_t, max_ was calculated to be 1.2, based on the meteorological and crop parameter data. Then, K_t_ [Equation (6)] was calculated based on Equation (5).


(6) $$\begin{array}{r} K_{t}=1.64NDVI-0.27 \end{array}$$

The above K_t_ represents the transpiration coefficient of cotton without any stress. However, in fact, the growth of cotton is affected by many factors, such as climate and agronomic management measures. Therefore, the coefficients of temperature stress (K_st_) and water stress (K_sw_) were introduced into the model according to the FAO-66 manual. The K_st_ was calculated according to Equation (7) (Raes et al., 2009).

**(2) Temperature stress coefficient (K**_**st**_ **)**


(7) $$K_{st}=\frac{S_{n}S_{x}}{S_{n}+(S_{x}-S_{n})\exp^{\left[-r(1-S_{relT}\right]}}$$

where S_n_ is the upper limit of K_st_, taken as 1, S_x_ is the lower limit of K_st_, taken as 0.001, S_relT_ is the relative water stress level [Equation (8)], and r is the ratio factor, taken as 15 (Venancio et al., 2019).


(8) $$\begin{array}{r} S_{relT}=\frac{T_{0}-T_{m}}{T_{0}-T_{b}} \end{array}$$

where T_0_ is the most suitable temperature for cotton growth (°C), taken as 30°C, T_b_ is the critical temperature (°C) for maintaining the normal growth of cotton, taken as 12°C, and T_m_ is the measured daily average temperature (°C). According to Steduto et al. (2012), when T_m_>T_0_, k_st_ = 1.

**(3) Water stress coefficient (K**_**sw**_ **)**

The water competition between shelterbelt and adjacent cotton plants may cause water stress on cotton and affect cotton B accumulation. The K_sw_ was calculated according to the following equation (Zhao et al., 2010):


(9) $$K_{sw}=\{\begin{array}{c} 1\theta \geq \theta J\\ \frac{\theta -\theta_{wp}}{\theta_{j}-\theta_{wp}}=\frac{\theta -\theta_{wp}}{\left(1-q)(\theta_{fc}-\theta_{wp}\right)}\theta_{wp}\leq \theta \leq \theta j \end{array}$$

where θ is the water content of the soil layer where most roots of crops are distributed (m^3^/m^3^), θ_fc_ is the water holding capacity of cotton field (m^3^/m^3^), taken as 25.4%, θ_wp_ is the wilting coefficient (m^3^/m^3^), taken as 11.2%, θ_j_ is the soil water content suitable for cotton growth (m^3^/m^3^), taken as 17.6%, and q is the ratio of crop water consumption to total available soil water without water stress (q = 0.55).

### Accuracy Evaluation and Model Verification

To verify the accuracy of the models in estimating WUE and WP, the measured data of two sampling plots at 0.1H, 1H, and 3H were used to construct models for estimating WUE and WP, and the data of the remaining one plot at 0.1H, 1H, and 3H and the plots at 0.5H and 2H were used as the verification set. The accuracy of the models was evaluated using the coefficient of determination (R^2^), root mean square error (RMSE), and consistency index (d) [Equation (10)]. Among them, d is a modified index for evaluating the accuracy based on the correlation (0 ≤ d ≤ 1). The larger the d, the higher the estimation accuracy.


(10) $$\begin{array}{l} \begin{array}{c} d=1-\frac{\sum_{i=1}^{n}{\left(Oi-Pi\right)}^{2}}{{\sum_{i=1}^{n}\left(|Pi-Ō|+|Oi-Ō|\right)}^{2}} \end{array} \end{array}$$

where O_i_ is the measured value, O_i_ is the average measured value, P_i_ is the estimated value, and N is the number of samples.

### Data Processing

The Excel software (version 2010, Microsoft, USA) was used for data processing and graphing, and the SPSS software (version 19, SPSS Inc., Chicago, USA) was used for the analysis of variance (*P* < 0.05) and significance test (Ducan).

## Results

### Meteorology and Soil Water Content in Different Growth Stages of Cotton

**Figure 3:**
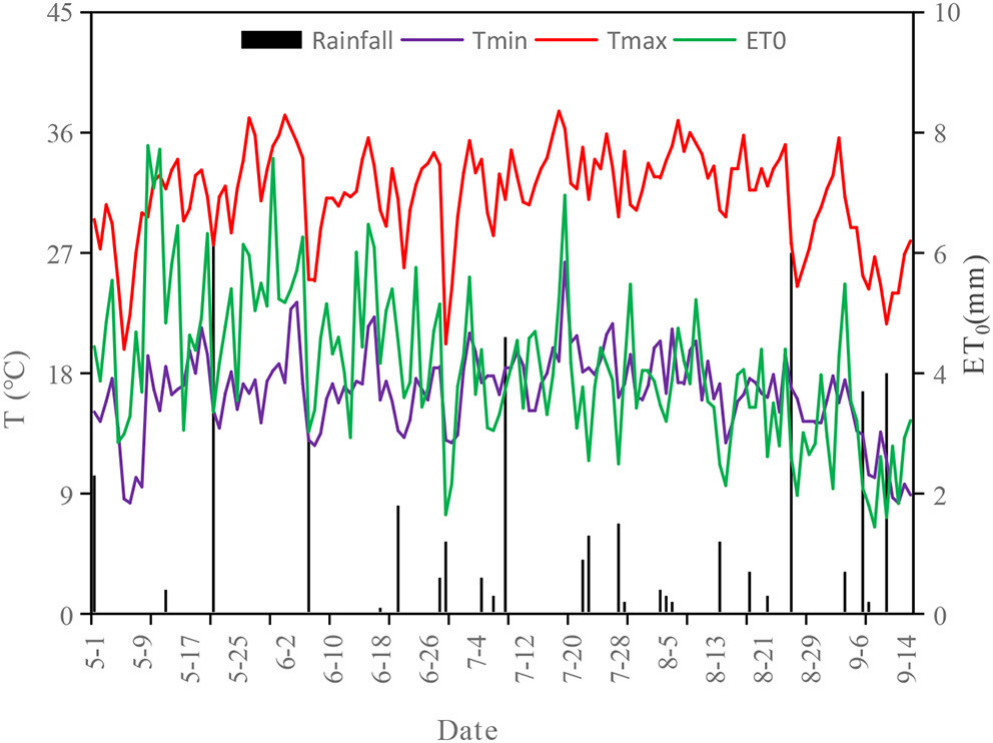
Variation in temperature and precipitation in the experimental site during the whole growth period of cotton.

During the entire growth period of cotton, the average temperature first increased and then decreased from May to September 2021. The ET_0_ showed a downward trend. From May to September, the monthly average temperature were 23.11, 23.89, 25.26, 24.42, and 19.75°C, respectively, the monthly precipitation were 8.9, 6.6, 9.4, 3.1, and 8.6 mm, respectively, and the monthly ET_0_ were 155.8, 136.0, 125.0, 108.9, and 41.9 mm, respectively. The ET_0_ in the whole growth period was 568 mm (Figure 3).

**Figure 4:**
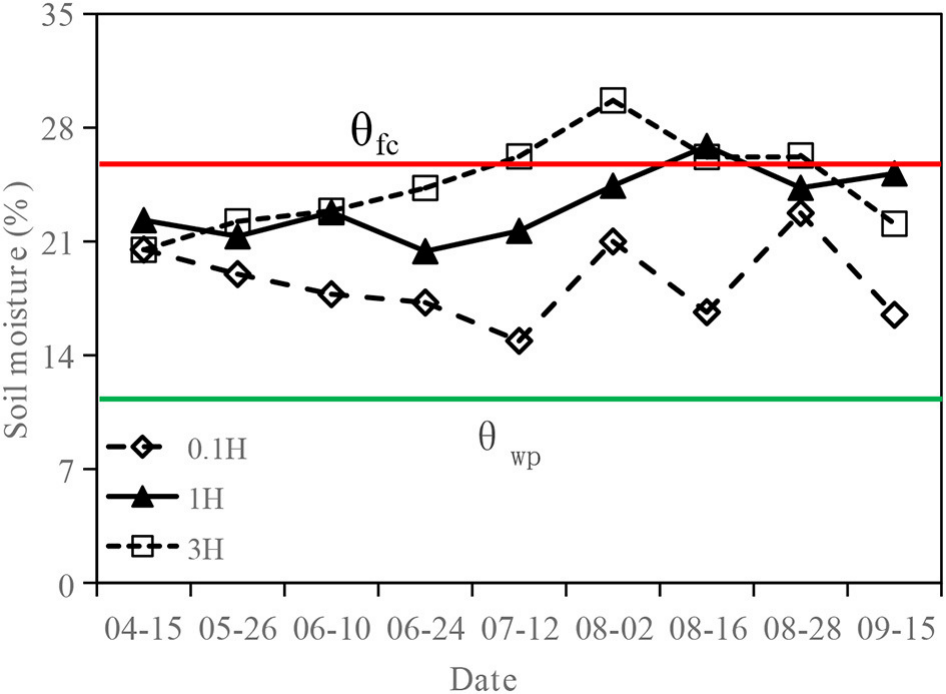
Variation of soil water content at different sampling areas (0.1H, 1H, and 3H).

Figure 4 shows the change of soil water content in cotton field. The soil water content at 0.1H was lower than that at 1H and 3H in the whole growth period (*P* < 0.05). The soil water content at 1H was higher than that at 3H from sowing to seedling stage, but lower than that at 3H from seedling stage to flowering stage (*P* < 0.05).

### Biomass and Normalized Difference Vegetation Index in Different Growth Stages of Cotton

**Figure 5:**
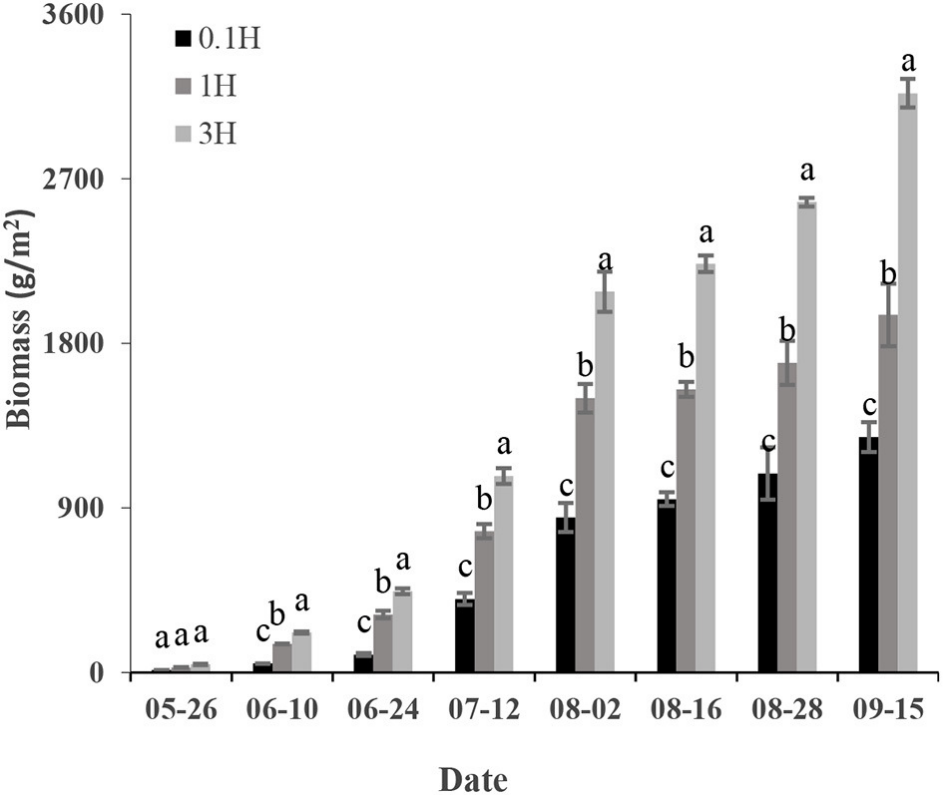
Variation of above-ground biomass of cotton in each sampling area (0.1H, 1H, and 3H).

In different growth stages, the changing trends of cotton B for the plots at different distances were the same. The cotton B gradually increased from May to September, and reached the maximum in the boll-opening stage. The cotton B at 3H was higher than that at 0.1H and 1H during the entire growth period of cotton (*P* < 0.05). In the boll opening stage, the cotton B at 0.1H and 1H decreased by 59 and 38%, respectively, compared with that at 3H (*P* < 0.05) (Figure 5).

**Figure 6:**
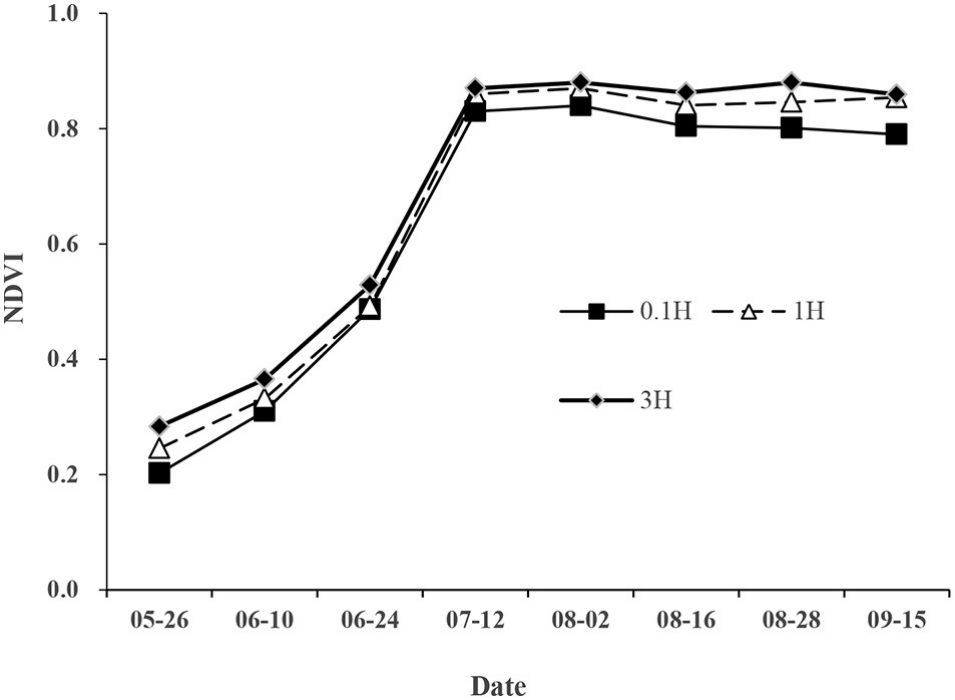
Changes of cotton Normalized difference vegetation index (NDVI) at 0.1H, 1H, and 3H.

The NDVI increased rapidly (5.26–7.12) at 0.1H, 1H, and 3H and then remained stable (8.2–9.15) (Figure 6). NDVI is related to the canopy coverage of cotton. Cotton leaves grew rapidly from the seedling stage to the flowering stage, so the canopy coverage and NDVI increased rapidly. Full coverage was achieved in the bud stage, and the NDVI reached the maximum value at this time (about 0.92). Cotton leaves began to wither, turn yellow, and fall off in the boll opening stage, resulting in a decrease in coverage and NDVI. The NDVI was the highest at 3H, followed by 1H and 0.1H (Figure 6).

### Variation of Coefficients of Water Stress and Temperature Stress

**Figure 7:**
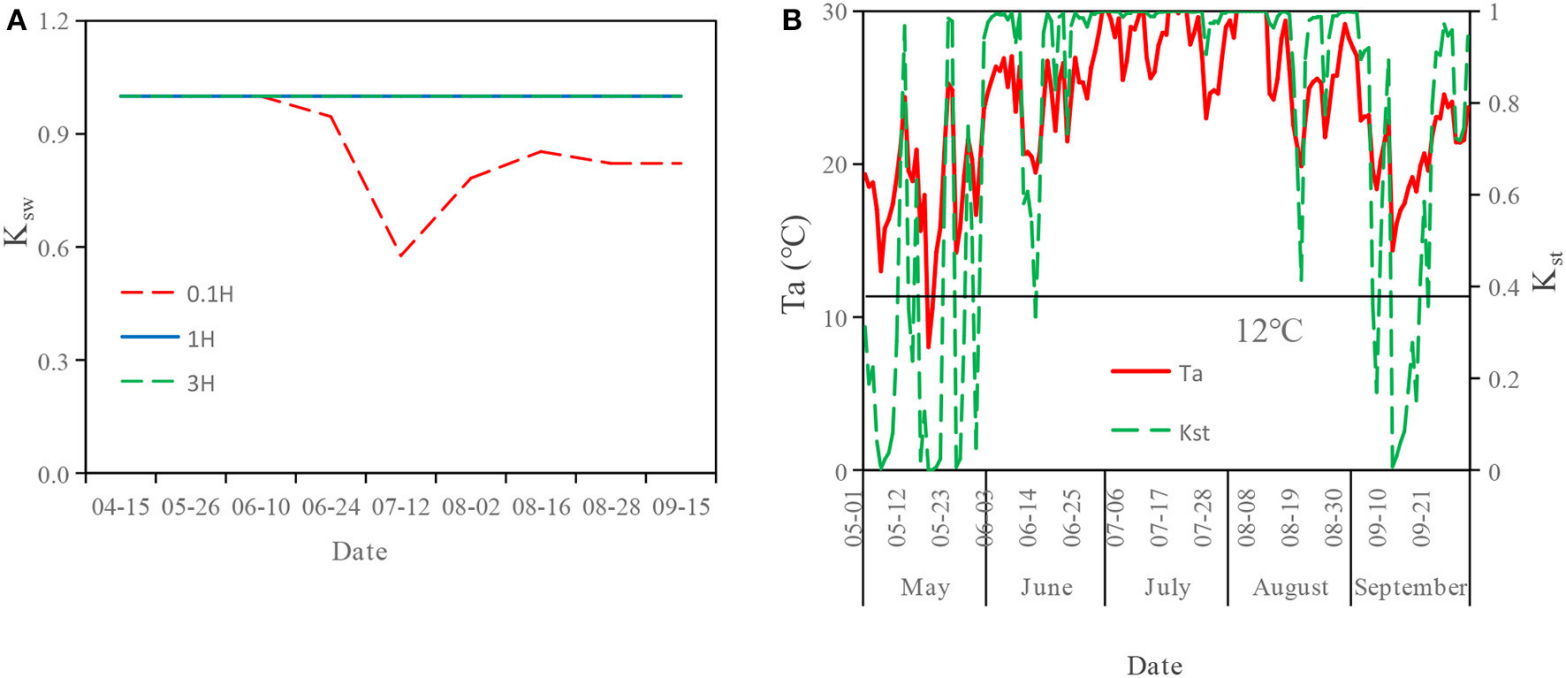
Changes of Water stress coefficient (K_sw_), Air temperature (Ta), and Temperature stress coefficient (K_st_) during the growth period of cotton at the sampling points with different distances from the shelterbelt. **(A)** represents water stress coefficient. **(B)** represents temperature stress coefficient.

There was no water stress for cotton plants at 0.1H, 1H, and 3H at the seedling stage, and the K_sw_ was equal to 1. However, due to the increased impacts of the shelterbelt in the bud stage, the K_sw_ at 0.1H was < 1. The average K_sw_ in the whole growth period at 0.1H, 1H, and 3H was 0.87, 1, and 1, respectively (Figure 7A).

The K_st_ was small in the seedling stage and boll-opening stage. The K_st_ in July was the maximum, followed by that in June and August. The daily average temperature was low in May and September. Therefore, the K_st_ was very small, close to 0 sometimes. The average K_st_ in the whole growth period was 0.75 (Figure 7B).

### Water Use of Cotton Field

#### Changes in WUE of Cotton During the Growth Period of Cotton

**Figure 8:**
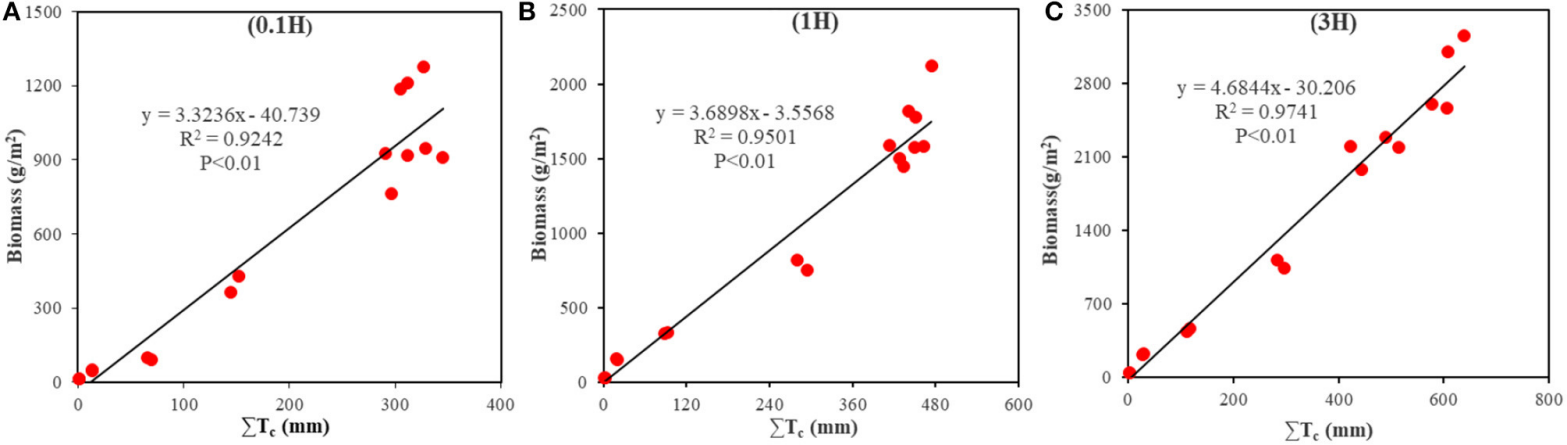
Fitting results of measured biomass and actual transpiration (T_c_) at the sampling points with different distances from the shelterbelt (0.1H, 1H, and 3H). **(A)** represents the fitting result within 0.1H. **(B)** represents the fitting result within 1H. **(C)** represents the fitting result within 3H.

Figure 8 shows the fitting results of measured B and estimated T_c_ at each growth stage of cotton. The results showed that there was a positive correlation between B and T_c_ at 0.1H, 1H, and 3H (*P* < 0.01), and the *R*^2^-values were 0.92, 0.95, and 0.97, respectively. The total water consumption in the whole growth period of cotton at 0.1H, 1H, and 3H was 311, 451, and 608 mm, respectively. In the analysis of WUE, the intercept of the linear relationship was not different from 0, so the slope was taken as WUE (Campos et al., 2018a,b). The WUE at 0.1H, 1H, and 3H was 3.3 (Figure 8A), 3.7 (Figure 8B), and 4.7 (Figure 8C) kg/m^3^, respectively.

The WUE at 0.1H, 1H, and 3H was obtained based on the measured B of harvested cotton and the T_c_ of the whole growth period estimated based on UAV remote sensing (Supplementary Figure S1). It can be seen that the average value of WUE and its coefficient of variation increased significantly with the increase of the distance from the shelterbelt. The average WUE values at 0.1H, 1H, and 3H showed that the WUE at 3H was the closest to the fitting result in Figure 8 (Table 1).

**Table 1:** Statistical analysis of water use efficiency (WUE) at the areas with different distances from the shelterbelt (0.1H, 1H, and 3H).

**Sampling area**	**Mean/(kg/m^**3**^)**	**Standard deviation/(kg/m^**3**^)**	**Coefficient of variation/(%)**
0.1H	3.89	0.58	14.91
1H	4.20	0.79	18.80
3H	5.09	0.98	19.25

#### Normalized Water Productivity

**Figure 9:**
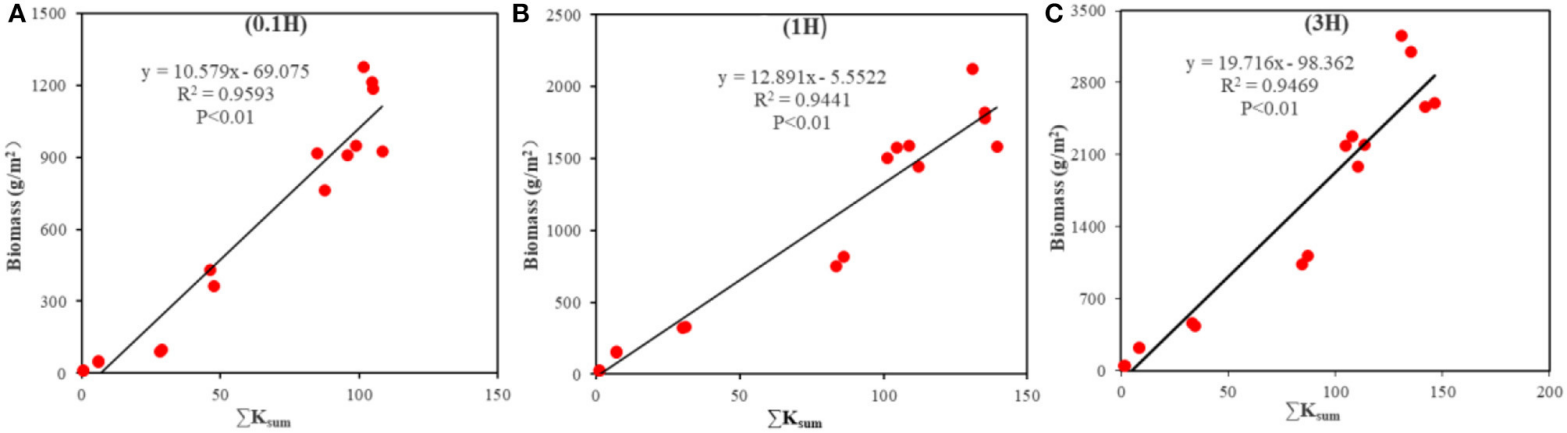
Correlation between biomass and K_sum_ at the sampling points with different distances from the shelterbelt (0.1H, 1H, and 3H). **(A)** represents the fitting result within 0.1H. **(B)** represents the fitting result within 1H. **(C)** represents the fitting result within 3H.

The WP and measured B had positive correlations with ΣK_sum_ (*P* < 0.01) (Figures 9A–C), and the *R*^2^-values were all >0.94. The WP values at 0.1H, 1H, and 3H were 10.6, 12.9, and 19.7 g/m^2^, respectively.

The WP was obtained based on the measured B and the K_sum_ in the whole growth period estimated based on UAV-based spectral data (Supplementary Figure S2). The WP increased significantly with the increase of the distance from shelterbelt. The mean values of WP at 0.1H, 1H, and 3H were 12.06, 14.67, and 23.89 g/m3, respectively (Table 2). The WP at 0.1H was closest to the fitting result, while the WP at 1H and 3H showed a relatively large difference to the fitting result (Figure 9). The mean value of WP changed significantly with the increase in the distance from shelterbelt. The farther the distance, the greater the WP. In contrast to WUE, the standard deviation of WP showed an increasing trend, while the coefficient of variation showed a decreasing trend. As shown in Table 2, the coefficients of variation of WUE at 0.1H, 1H, and 3H were 23.05, 20.17, and 15.45%, respectively.

**Table 2:** Statistical analysis of normalized water productivity (WP) of cotton plants at different areas (0.1H, 1H, and 3H).

**Sampling area**	**Mean (g/m^**2**^)**	**Standard deviation (g/m^**2**^)**	**Coefficient of variation (%)**
0.1H	12.06	2.78	23.05
1H	14.67	2.96	20.17
3H	23.89	3.69	15.45

### Model Validation and Biomass Estimation

**Figure 10:**
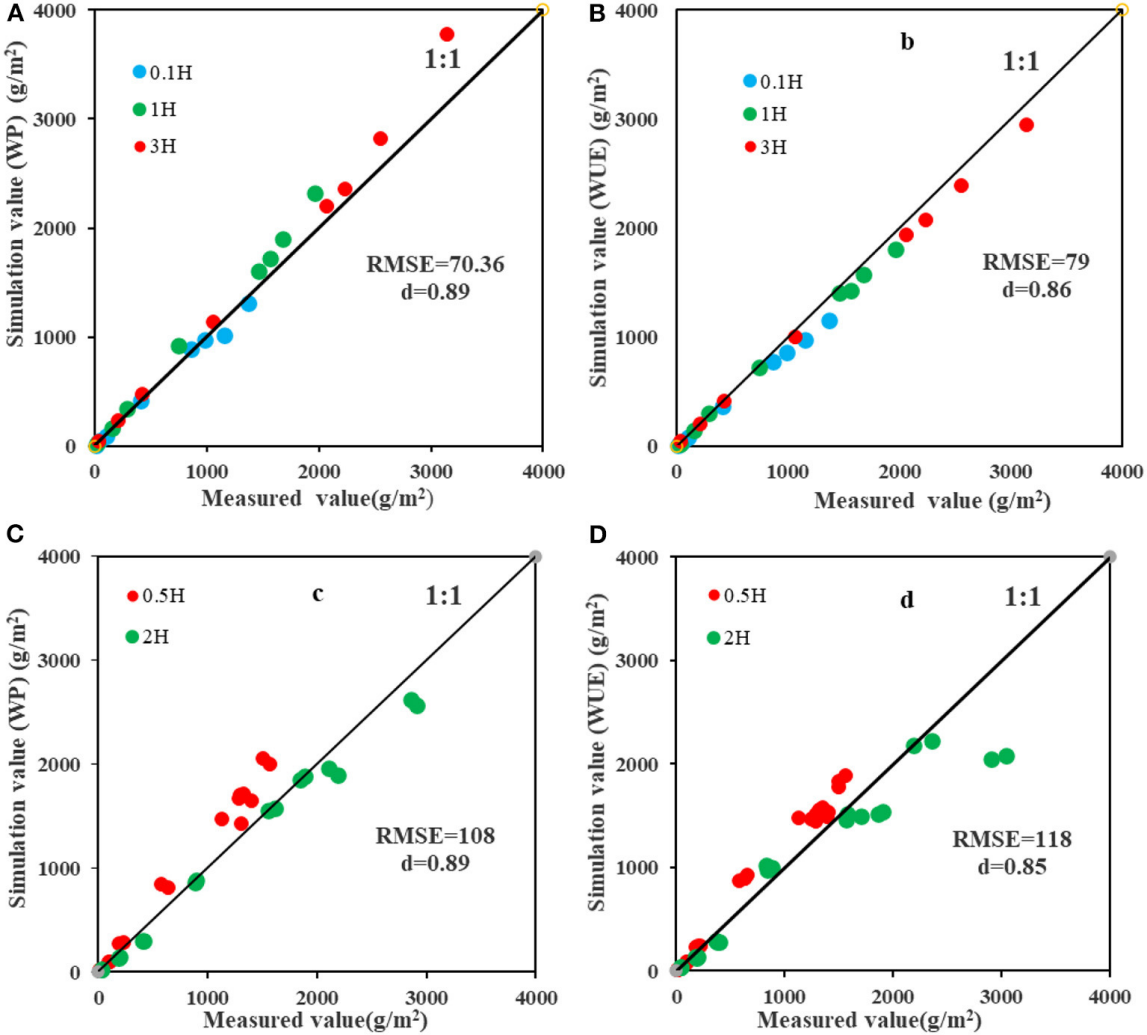
Analysis of the estimation accuracy of the measured and estimated biomass at the sampling areas with different distances from the shelterbelt (0.1H, 0.5H, 1H, 2H, and 3H). **(A)** Fitting result between measured biomass and estimated biomass based on the estimated WP at 0.1H, 1H, and 3H. **(B)** Fitting result between measured and estimated biomass based on the estimated WUE at 0.1H, 1H, and 3H. **(C)** Fitting result between measured and estimated biomass based on the estimated WP at 0.5H and 2H. **(D)** Fitting result between measured and estimated biomass based on the estimated WUE at 0.5H and 2H.

The WUE and WP fitted in the “Estimation of cotton biomass based on UAV-based multispectral remote sensing and AquaCrop model” section were combined with Equation (1) and (2) to estimate the B at 0.1H, 1H, and 3H, and the estimation results were verified by the measured B (Figures 10A,B). It could be seen that the estimation accuracy based on the estimated WP (RMSE = 70.36 g/m^2^, d = 0.89) was higher than that based on the estimated WUE in the whole growth period.

There were differences in the impacts of the shelterbelt on the water use of cotton plants at different distances from shelterbelt. To further verify the universality of the model, the average values of WUE and WP at 0.1H, 1H, and 3H (3.9 g/m^2^ and 14.4 g/m^2^, respectively) were separately used to estimate the B at 0.5H and 2H in different growth stages except for the harvest stage, and the accuracy was verified by the measured B at 0.5H and 2H (Figures 10C,D). The results showed that the B estimated based on the average WUE was overestimated at 0.5H and underestimated at 2H. The accuracy in estimating B based on WP was higher than that based on WUE. However, when WP was used for estimation, it was found that when measured B was <2,000 g/m^2^, the estimation was more accurate at 2H than at other distances, and overestimation occurred at 0.5H. When measured B was >2,000 g/m^2^, the B at 2H was underestimated. In general, the accuracy in estimating B based on WP (RMSE = 108 g/m^2^, d = 0.89) was higher than that based on WUE (RMSE = 118 g/m^2^, d = 0.85).

### Spatial Distribution of Biomass

According to the results in the “Accuracy evaluation and model verification” section, the estimation accuracy of B based on WP was higher than that based on WUE. Therefore, Equation 2 was used to estimate the B of harvested cotton at 0.5H and 2H. The K_sw_ at 0.5H and 2H was 1. There was a significant difference in the average B between 0.5H (1,384 g/m^2^) and 2H (2,082 g/m^2^) (Supplementary Figure S3).

## Discussion

### Relationship in Water Use Between Tree and Cotton in the Farmland-Shelterbelt System

Some studies have explored the impacts of shelterbelt on the water use of adjacent crops under flood irrigation (Nie et al., 2019; Hu and Jia, 2021). However, in this study, the impacts of the shelterbelt on the water use of adjacent crops were explored under drip irrigation. It was found that the soil water content at 0.1H was lower than that at 1H and 3H in the whole growth period, indicating that the farther away from the shelterbelt, the higher the soil water content. This is similar to the study results of Yin et al. (2011), Hu and Jia (2021), and Wu et al. (2021). In addition, the average K_sw_ at 0.1H was <1 in the whole growth period. This indicates that the cotton plants at 0.1H were subject to certain water stress caused by the shelterbelt. The average K_sw_ values at 1H and 3H were equal to 1 in the whole growth period. This indicates that the shelterbelt had less effect on the water use of cotton within 1H−3H. The variation of soil water content and K_sw_ all showed that there was a water competition between shelterbelt and cotton in 0.1H−1H under drip irrigation. At 2H and 3H (>1H), the influence of the shelterbelt on cotton began to weaken. The previous study has shown that poplars compete with cotton plants for soil water through extending lateral roots into the cotton field, with an impact ranging from 0 to 8 m in the cotton field (Guo et al., 2019). However, in this study, in the bud, bolling, and boll-opening stage, with the growth of cotton, the range under the impact of shelterbelt decreased. This is consistent with the study results of Judd and McAnarney (1992) and Nie et al. (2019).

### Effects of Shelterbelt on WUE, Evapotranspiration, and Biomass of Adjacent Cotton Plants

Water is one of the key factors influencing root distribution and interspecific competition in agroforestry ecosystems. The interspecific relationship in water use plays a decisive role in the productivity of agroforestry ecosystems in an oasis. Based on the study by Han et al. (2021), multispectral UAV images were used in this study to estimate the growth status of cotton near the shelterbelt in different growth stages of cotton. Based on the meteorological data, soil water content, and AquaCrop model, the WUE and WP of cotton plants with different distances from the shelterbelt were estimated accurately. The estimation error using the UAV-based remote sensing in this study was smaller than that based on the satellite remote sensing by Campos et al. (2018a) and Venancio et al. (2019). This study found that the farther away from the shelterbelt, the greater the WUE and WP, indicating that the farther away from the shelterbelt, the weaker the water stress on cotton. Besides, this study found that the WUE and WP at 3H were 42.4 and 85.8% higher than that at 0.1H, respectively (Figures 8, 9). This is consistent with the study results of Lian et al. (2021) and He et al. (2016). It shows that the estimation method adopted by this study can clarify the impacts of shelterbelt on the WUE of adjacent cotton plants. Moreover, in this study, the evapotranspiration at 3H was more than that at other distances (*P* < 0.05). This may be one of the reasons why the cotton plants at 3H are not affected by the shelterbelt. The cooling effect of shelterbelt has been demonstrated in previous studies (Mao et al., 2018). In this study, shelterbelts had no impact on the cotton plants at 3H. Therefore, the cotton plants at 3H are exposed to intense solar radiation, leading to the higher temperature and lower relative humidity at 3H. Under these conditions, the stomata of cotton leaves open, and the stomatal conductance, photosynthetic rate, and transpiration rate are higher than those of cotton plants under the impact of shelterbelt (Sun et al., 2018).

The spectral features of the cotton canopy obtained by UAV could reflect cotton growth status. The AquaCrop model combined with the spectral features can accurately estimate the water use of cotton in each growth stage (Figures 8, 9). The evapotranspiration values of cotton at the bud stage, bolling stage, and boll-opening stage at 3H were 81, 322, and 31 mm, respectively, which were 17–20% different from that estimated using the Eddy-covariance method (Ma et al., 2015). This shows that estimating the evapotranspiration by using the method proposed in this study is feasible.

Water is the most important factor influencing plant growth in an oasis (Ong et al., 2002; Wang et al., 2018). Water competition between shelterbelt and cotton is one of the main reasons for the yield decline of cotton plants adjacent to the shelterbelt (Hu and Jia, 2021). Therefore, accurate estimation of B of cotton with different distances from the shelterbelt can clarify the impact range and degree of shelterbelt on cotton. June–August is the vigorous growth period of cotton canopy. In this study, the NDVI at 3H was greater than that at 1H and 0.1H (*P* < 0.05). This indicates that shelterbelt has a certain effect on the NDVI of an adjacent cotton field. Besides, in this study, the B at 2H was 50.4% higher than that at 0.5H (*P* < 0.05). This is consistent with the study results of Zhu et al. (2010). It indicates that shelterbelt has a certain negative effect on the B of adjacent cotton plants, and the estimation method adopted in this study is feasible and has a high accuracy (RMSE = 108 g/m^2^, d = 0.89). Many studies used remote sensing data to estimate crop B at a certain crop growth stage. For example, Sindhuja et al. (2018) used green NDVI (GNDVI) to estimate the B of dry bean in two growth stages, finding that the highest R^2^ was 0.73. In this study, the R^2^ of the AquaCrop model constructed based on UAV remote sensing data and measured data in estimating cotton B reached 0.92, which was higher than that of the models in previous studies.

### Model Selection and Error Analysis

In this study, the AquaCrop model was used to calculate WUE and B under drip irrigation (Campos et al., 2018a,b; Venancio et al., 2019). The impacts of shelterbelt on WUE and B of adjacent cotton were explored based on UAV-based remote sensing and AquaCrop model. Plots with different distances from the shelterbelt were set in the cotton field, and the B estimated based on WUE and WP was verified by using the measured B. It was found that the estimated B based on WP was more accurate than that based on WUE (Figures 8, 9). Besides, the estimated B based on WUE and WP had high accuracy in the seedling stage, bud stage, and flowering and boll-forming stage, but the estimation accuracy based on WUE decreased in boll-opening stage. This is similar to the study results of Campos et al. (2018a); Deng et al. (2019), and Han et al. (2021). Therefore, it can be inferred that shelterbelt has a great impact on the WUE of adjacent cotton plants by water competition. Besides, using WUE to estimate cotton B will cause a large error. However, when the measured B at 0.5H and 2H were used to verify the B estimated based on WUE and WP at 0.1H, 1H, and 3H (Figures 8, 10), it was found that the accuracy in estimating B based on WP was higher (Figures 10C,D). Besides, the Bs estimated based on WP and WUE were overestimated at 0.5H and underestimated at 2H. This may be due to that the WP at 1H and 3H was higher than that at 0.5H, and the average value of the WUE at 0.1H, 1H, and 3H was used for the estimation, leading to the overestimated B at 0.5H. Besides, due to the WUE at 0.1H and 1H was lower than that at 2H, the average value of the WUE at 0.1H, 1H, and 3H was adopted for the estimation, and the B at 2H was underestimated. Mutanga and Skidmore (2004) and Zheng et al. (2017) showed that underestimation of B might be related to the NDVI. In this study, due to the full coverage of cotton canopy, the sensitivity of NDVI to the change of B may reduce in the later growth stage of cotton, which affects the estimation accuracy of WUE. Han et al. (2021) believed that since ΣT_c_ and ΣK_sum_ were increasing in the model, the growth rate of B decreased with the decrease of K_sw_. In fact, the B growth rate of crops with strong drought tolerance will not decline rapidly. However, in this study, it was assumed that the B growth rate declined rapidly, so there was an underestimation. To sum up, the WUEs of cotton plants with different distances from the shelterbelt are different due to the difference in the intensity of competition. Therefore, using WP to estimate the B of cotton with different distances from the shelterbelt has higher accuracy than using WUE. However, due to the underestimation of B in some growth stages of cotton, some researchers have proposed that a non-linear model could be used to replace the AquaCrop model to improve estimation accuracy (Ran et al., 2019; Han et al., 2021). Therefore, the non-linear model for estimating WUE or WP based on the UAV-based remote sensing will be the focus of our future study.

## Conclusion

Based on UAV remote sensing data, AquaCrop crop growth model, meteorological data, soil moisture content, and other measured crop data, this study accurately simulates the spatial distribution of WUE, WP, and B of cotton plants with different distances from shelterbelt in each growth stage of cotton. Compared with conventional methods, our method can more accurately estimate the WUE, water consumption, and B of cotton plants adjacent to the shelterbelt. Besides, the impact range and intensity of shelterbelt on adjacent cotton plants can also be accurately quantified. In our study, the shelterbelt mainly affected the cotton plants with a distance of <1H from the shelterbelt. Especially at 0.1H, the cotton B was reduced by more than half. However, the cotton plants at 2H and 3H were completely unaffected by the shelterbelt. Therefore, additional irrigation should be carried out for adjacent cotton plants to reduce the negative impact of the shelterbelt. Besides, the estimation of cotton B based on WP was more accurate than that based on WUE. The higher error in estimating cotton B based on WUE is caused by the low accuracy of the simulated WUE value. The linear model used in this study led to the underestimation of B in the later stage of cotton growth. Therefore, combining non-linear models with UAV remote sensing data to estimate WUE or WP is the focus of our next study.

## Data Availability Statement

The original contributions presented in the study are included in the article/Supplementary Material, further inquiries can be directed to the corresponding author/s.

## Author Contributions

YJ: conceptualization (lead), funding acquisition (lead), methodology (equal), and writing–review and editing (supporting). BM: investigation (equal), methodology (supporting), writing–original draft (lead), and writing–review and editing (lead). QW: data curation (lead), formal analysis (supporting), and methodology (supporting). BX: resources (supporting) and data curation (supporting). ZH: formal analysis (lead), methodology (equal), project administration (lead), resources (lead), and supervision (lead). WC: formal analysis (lead), methodology (equal), and resources (lead). All authors contributed to the article and approved the submitted version.

## Funding

This research was supported by the National Natural Science Foundation of China (31660135) and the Independent Project of Shihezi University (KX03100304).

## Conflict of Interest

The authors declare that the research was conducted in the absence of any commercial or financial relationships that could be construed as a potential conflict of interest.

## Publisher's Note

All claims expressed in this article are solely those of the authors and do not necessarily represent those of their affiliated organizations, or those of the publisher, the editors and the reviewers. Any product that may be evaluated in this article, or claim that may be made by its manufacturer, is not guaranteed or endorsed by the publisher.

## Abbreviations

H: average tree height (15 m)
WUE: Water use efficiency
WP: Normalized water productivity
ET_0_: Standardized reference evapotranspiration
NDVI: Normalized difference vegetation index
NDVI_max_: Maximum NDVI when the canopy coverage of cotton is the maximum
NDVI_min_: NDVI based on bare soil
UAV: Unmanned aerial vehicle
T_c_: Actual evapotranspiration
K_t_: Transpiration coefficient
K_st_: Temperature stress coefficient
K_sw_: Water stress coefficient
K_sum_, Product of K_st_, K_sw_: and K_t_
B: Cotton biomass
t_0_: Emergence time of cotton
t_1_: Time of biomass data acquisition
K_t, max_: Maximum transpiration coefficient
T_0_: The most suitable temperature for cotton growth
T_b_: The critical temperature (°C) for maintaining the normal growth of cotton
T_m_: Daily average temperature
θ: Water content of the soil layer where crop root most distributed
θ_fc_: Water holding capacity of cotton field
θ_wp_: Wilting coefficient
θ_j_: The soil water content suitable for cotton growth
d: Consistency index
S_n_: The upper limit of temperature stress coefficient
S_x_: The lower limit of temperature stress coefficient
S_relT_: Relative level of water stress
r: Ratio factor.
