# Supplementary material for: Application of UAV Remote Sensing in Monitoring Water Use Efficiency and Biomass of Cotton Plants Adjacent to Shelterbelt
Source: Front Plant Sci. 2022 Jun 16;13:894172. doi: 10.3389/fpls.2022.894172 (PMC9244790; doi:10.3389/fpls.2022.894172)
Supplement: Supplementary file 1 [file Data_Sheet_1.docx]

Fig. S1 Water use efficiency (WUE) of cotton in the whole growth period at the sampling points with different distances from the shelterbelt (0.1H, 1H, and 3H)

Fig. S2 Distribution of normalized water productivity (WP) in the whole growth period of cotton plants with different distances from the shelterbelt (0.1H, 1H, and 3H)

Fig. S3 Biomass of cotton plants at 0.5H and 2H estimated based on the mean normalized water productivity (WP)

Fig. S1 Water use efficiency (WUE) of cotton in the whole growth period at the sampling points with different distances from the shelterbelt (0.1H, 1H, and 3H)


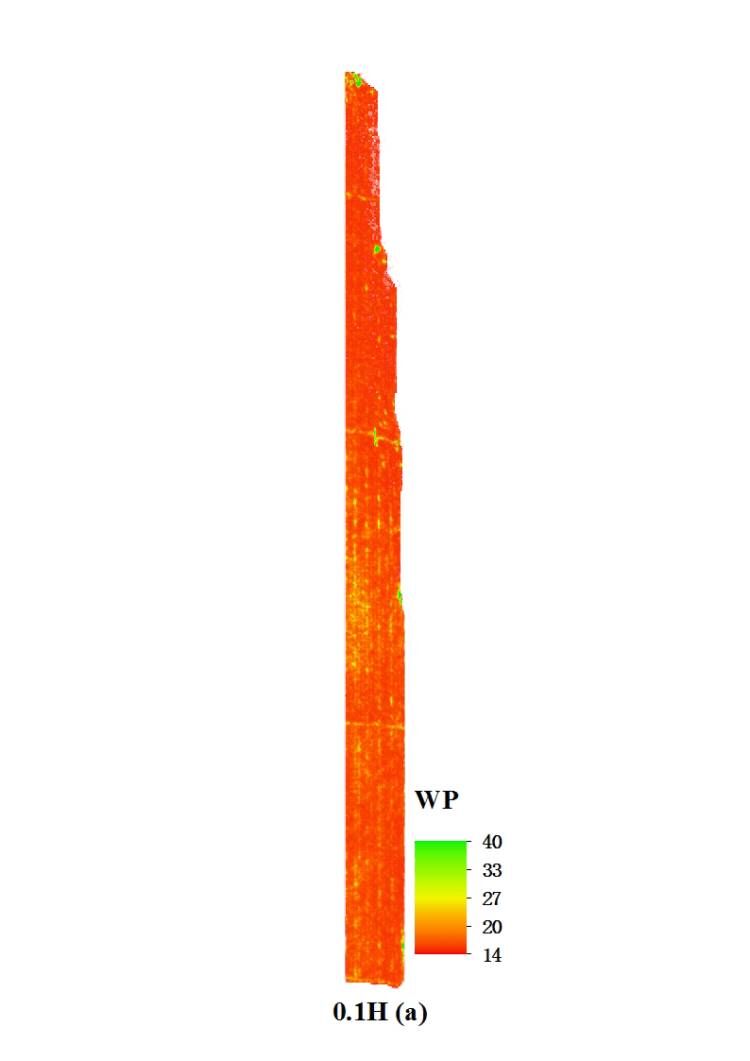

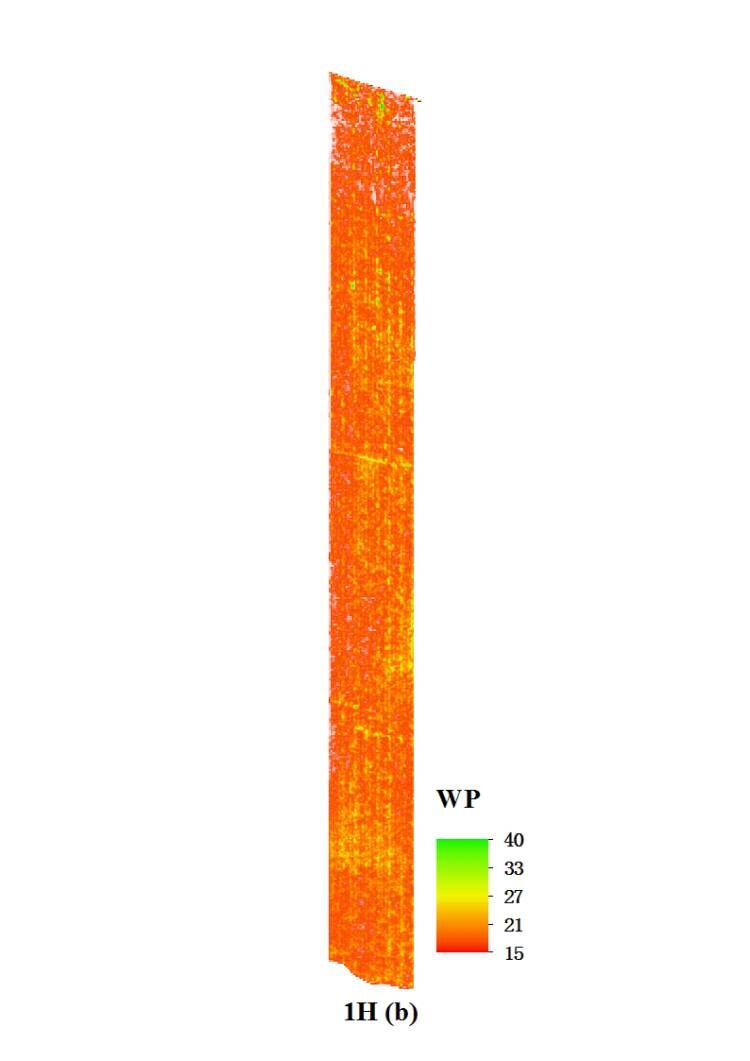

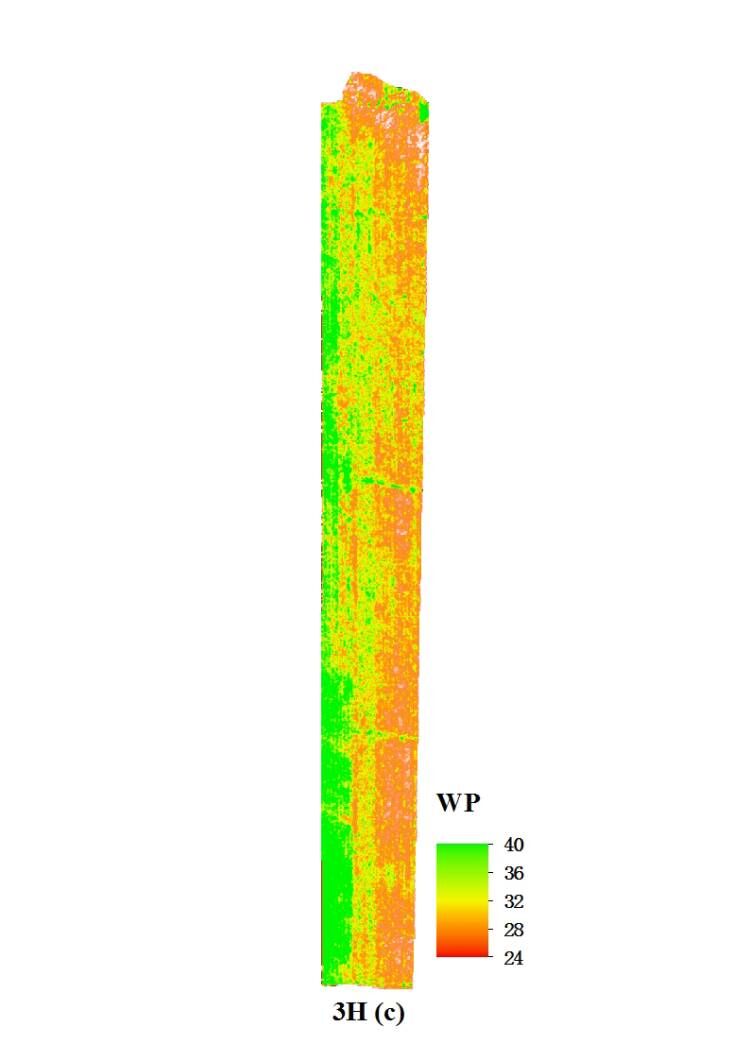


Fig. S2 Distribution of normalized water productivity (WP) in the whole growth period of cotton plants with different distances from the shelterbelt (0.1H, 1H, and 3H)


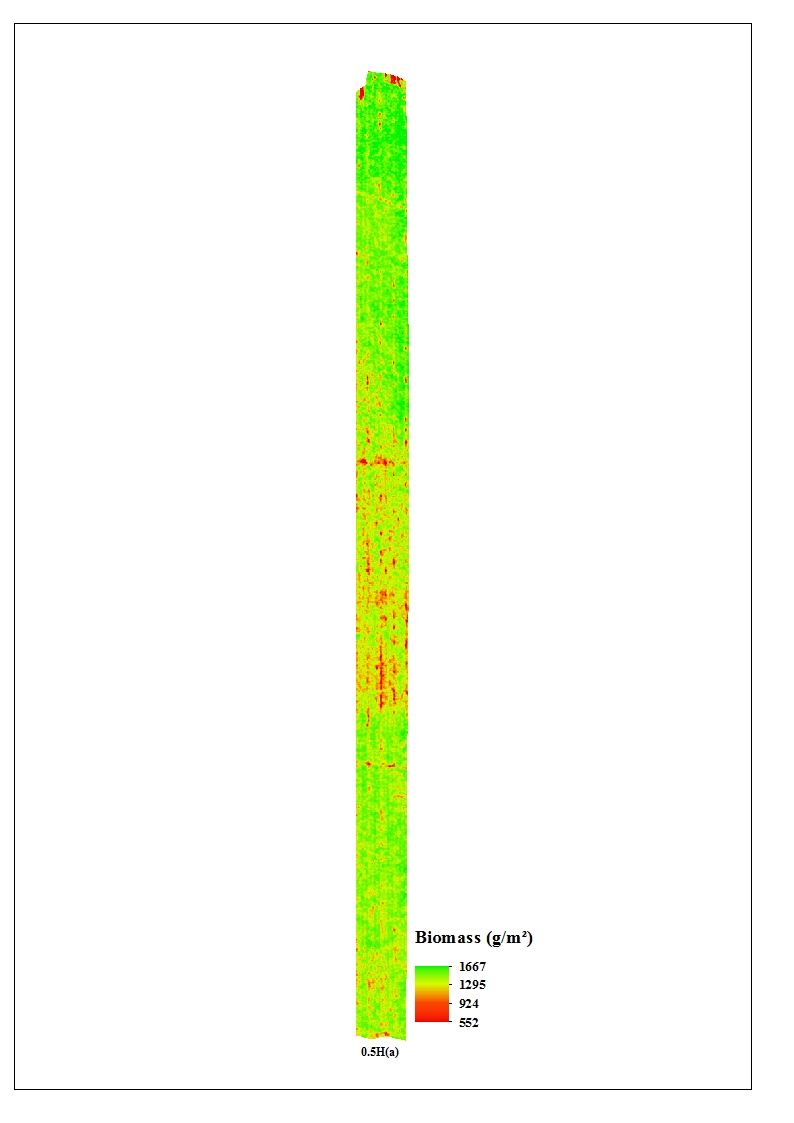

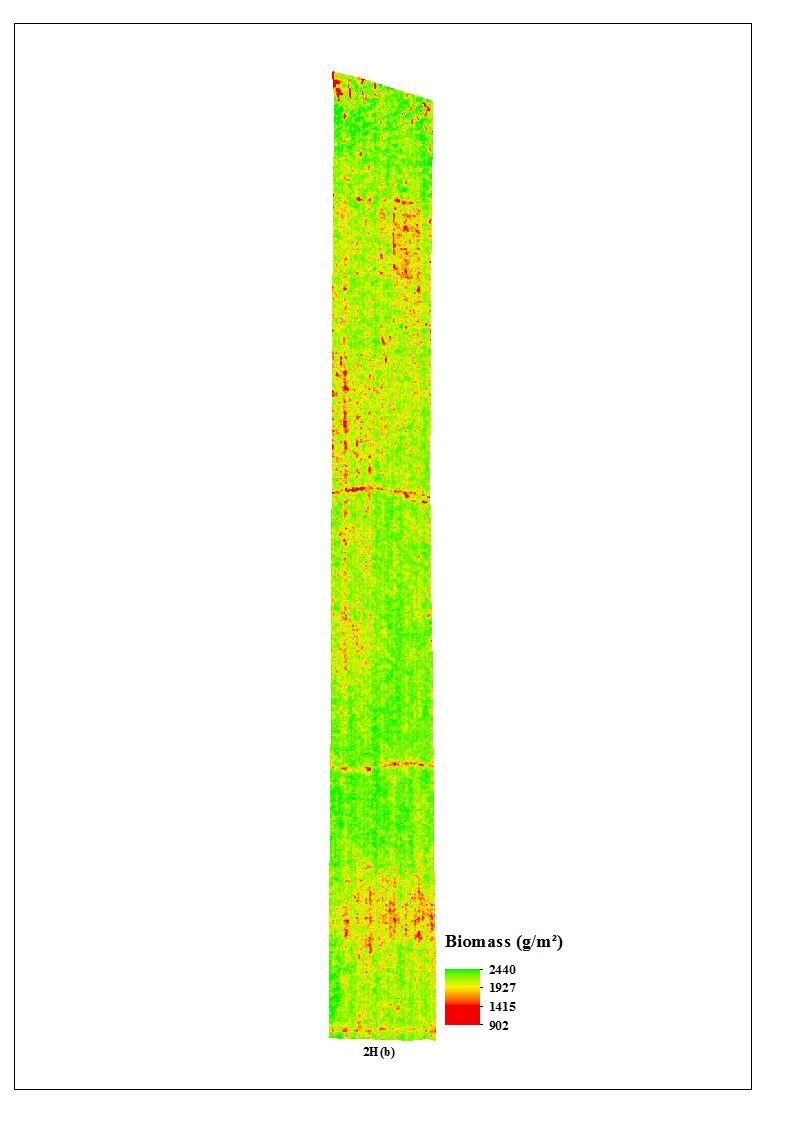


Fig. S3 Biomass of cotton plants at 0.5H and 2H estimated based on the mean normalized water productivity (WP)
